# Graves’ disease in a mediastinal mass presenting after total thyroidectomy for nontoxic multinodular goiter: a case report

**DOI:** 10.1186/s13256-016-0878-7

**Published:** 2016-03-31

**Authors:** Filipe Manuel Cunha, Elisabete Rodrigues, Joana Oliveira, Ana Saavedra, Luís Sá Vinhas, Davide Carvalho

**Affiliations:** 1 Serviço de Endocrinologia, Diabetes e Metabolismo, Centro Hospitalar de São João, Alameda Professor Hernâni Monteiro, 4202-451 Porto, Portugal; 2 Faculdade de Medicina da Universidade do Porto, Porto, Portugal; 3 Serviço de Cirurgia Geral, Centro Hospitalar de São João, Porto, Portugal

**Keywords:** Graves’ disease, Mediastinal mass, Hyperthyroidism, Total thyroidectomy, Thyroid remnant

## Abstract

**Background:**

Thyrotoxicosis after total thyroidectomy is mostly iatrogenic. Rarely, a hyperfunctional thyroid remnant or ectopic tissue may be the cause. There are few cases of Graves’ disease arising from thyroid tissue located in the mediastinum and none in which Graves’ disease was diagnosed only after surgery. We report the case of a patient with Graves’s disease in a mediastinal thyroid mass presenting 7 years after total thyroidectomy for nontoxic goiter.

**Case presentation:**

A 67-year-old Caucasian woman presented with palpitations, fatigue and weight loss. She had a history of total thyroidectomy for nontoxic multinodular goiter at the age of 60 without any signs of malignancy on microscopic examination. She had been medicated with levothyroxine 100 μg/day since the surgery without follow-up. She was tachycardic, had no cervical mass or eye involvement. Her thyroid-stimulating hormone levels were suppressed (0.000 μU/mL) and her free thyroxine (3.22 ng/dL) and free triiodothyronine (8.46 pg/mL) levels increased. Neither mediastinal enlargement nor trachea deviation was found on chest roentgenogram. Levothyroxine treatment was stopped but our patient showed no improvement on free thyroxine or free triiodothyronine 10 days later. Thyroglobulin was increased to 294 mg/mL. A cervical ultrasound scan revealed no thyroid remnant. Her anti-thyroid-stimulating hormone receptor antibodies were high (19.7 U/L). Corporal scintigraphy demonstrated increased intrathoracic radioiodine uptake. A computed tomography scan confirmed a 60 × 40 mm mediastinal mass. Methimazole 10 mg/day was started. Three months later, her thyroid function was normal and she underwent surgical resection. Microscopic examination showed thyroid tissue with no signs of malignancy.

**Conclusions:**

Although thyrotoxicosis after total thyroidectomy is mostly due to excessive supplementation, true hyperthyroidism may rarely be the cause, which should be kept in mind. The presence of thyroid tissue after total thyroidectomy in our patient may correspond to a remnant or ectopic thyroid tissue that became hyperfunctional in the presence of anti- thyroid-stimulating hormone receptor antibodies.

## Background

Graves’ disease is the most common cause of hyperthyroidism [1]. Thyrotoxicosis after total thyroidectomy is mainly due to supratherapeutic doses of levothyroxine; other possible, although more rare etiologies are a hyperfunctional thyroid remnant or ectopic thyroid tissue [2].

The presence of a thyroid remnant after total thyroidectomy is not infrequent. When total thyroidectomy is performed for benign pathology, the estimated recurrence rate is 0–0.3 % [3, 4]. This possibly represents an underestimation since only symptomatic recurrence was detected. If ultrasonography is performed to detect the presence of a thyroid remnant, the recurrence rate can reach 33 % [5]. Recurrence is mostly due to the presence of an embryologic thyroid remnant along the pyramidal or thyrothymic tract that can be missed during surgery [4].

The existence of ectopic thyroid tissue is a rare disorder that results from an abnormal migration of the thyroid gland during embryological development. It is not a frequently encountered entity in everyday clinical practice – prevalence of 1:100,000 to 1:300,000 – but in autopsy studies its prevalence is higher – from 7–10 % [6, 7]. It appears that the existence of ectopic thyroid tissue is relatively common, but most of the time asymptomatic and thus undetected. The ectopic thyroid tissue can be affected by the same diseases that involve the thyroid gland [6, 7]. An intrathoracic location of thyroid tissue is uncommon and it usually coexists with a normally functioning thyroid gland. An intrathoracic ectopic thyroid may be asymptomatic or present with compressive symptoms [6].

We present the case of a 67-year-old woman previously submitted to total thyroidectomy for a multinodular nontoxic goiter who presented with hyperthyroidism.

## Case presentation

A 67-year-old Caucasian woman was admitted to the emergency department with palpitations, and dizziness in the past 2 days. She complained of fatigue after minor effort, weight loss (11 kg in 3 years) without anorexia, and hand tremor. She had no complaints of dyspnea, orthopnea or chest pain. She had no heat intolerance nor increased frequency of bowel movements.

Our patient had a history of breast cancer diagnosed at the age of 37 and treated with right radical mastectomy and adjuvant chemotherapy. She underwent total thyroidectomy for nontoxic multinodular goiter [thyroid-stimulating hormone (TSH) 3.77 of μU/mL and free thyroxine (T4) of 1.53 ng/dL] with a dominant colloid nodule of 30 mm at the age of 60; the histological analysis confirmed multinodular adenomatous goiter weighing 66 g with no signs of malignancy. She was medicated with levothyroxine 100 μg/day since the surgery and had no follow-up. She smoked 20 cigarettes a day for the last 50 years.

At physical examination, our patient was conscious and oriented. Her temperature was 36.7 °C, she had a body mass index of 19.7 kg/m^2^, blood pressure of 122/76 mmHg, a heart rate of 130 bpm, and fine tremor at rest. She had no eye involvement. A cervical examination showed no palpable masses or adenomegaly. Pemberton’s sign was negative. The pulmonary sounds were normal and she had no cardiac murmur. She had no peripheral edema.

At admission, our patient had a hemoglobin level of 11.5 g/L (12–16); a normal white blood cell and platelet count; a normal liver panel; an albumin level of 32.6 g/dL (38–51); normal renal function and ionogram; corrected total calcium level of 9.6 mg/dL (8.1–10.4); B-type natriuretic peptide level of 442.2 pg/mL (<100); and a troponin I level <0.010 ng/mL. She had suppressed levels of TSH (0.000 μU/mL) and increased levels of free T4 3.22 ng/dL (0.70–1.48) and free triiodothyronine (T3) 8.46 pg/mL (1.71–3.71). In the electrocardiogram, she had sinus tachycardia of 117 bpm and incomplete right bundle branch block and left anterior fascicle block. Her chest roentgenogram showed normal cardiothoracic index, no pulmonary congestion, no mediastinal enlargement nor tracheal deviation.

**Fig. 1:**
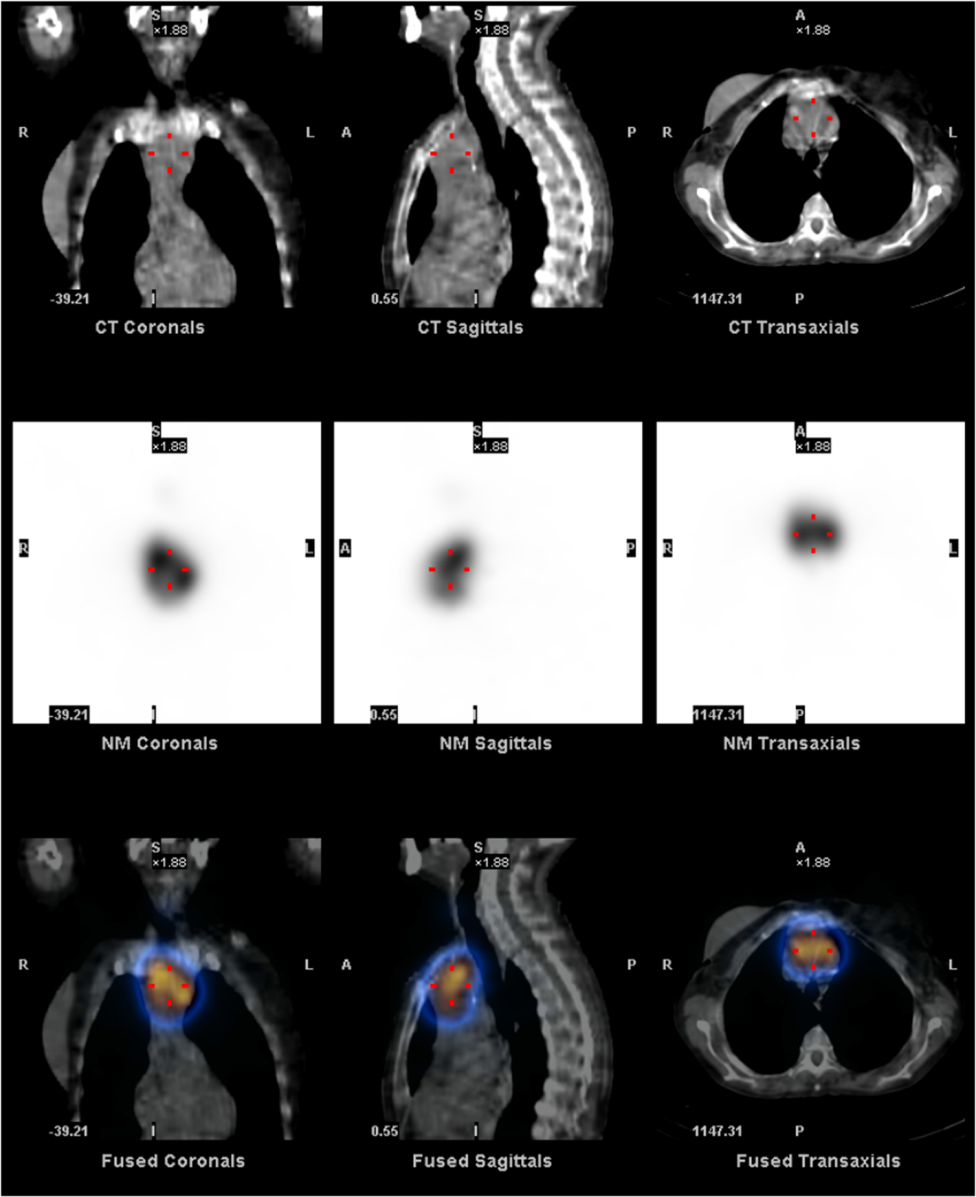
Corporal scintigraphy with I-131 with single-photon emission computed tomography showing an intrathoracic mass (60 × 40 mm) with increased radioiodine uptake

**Fig. 2:**
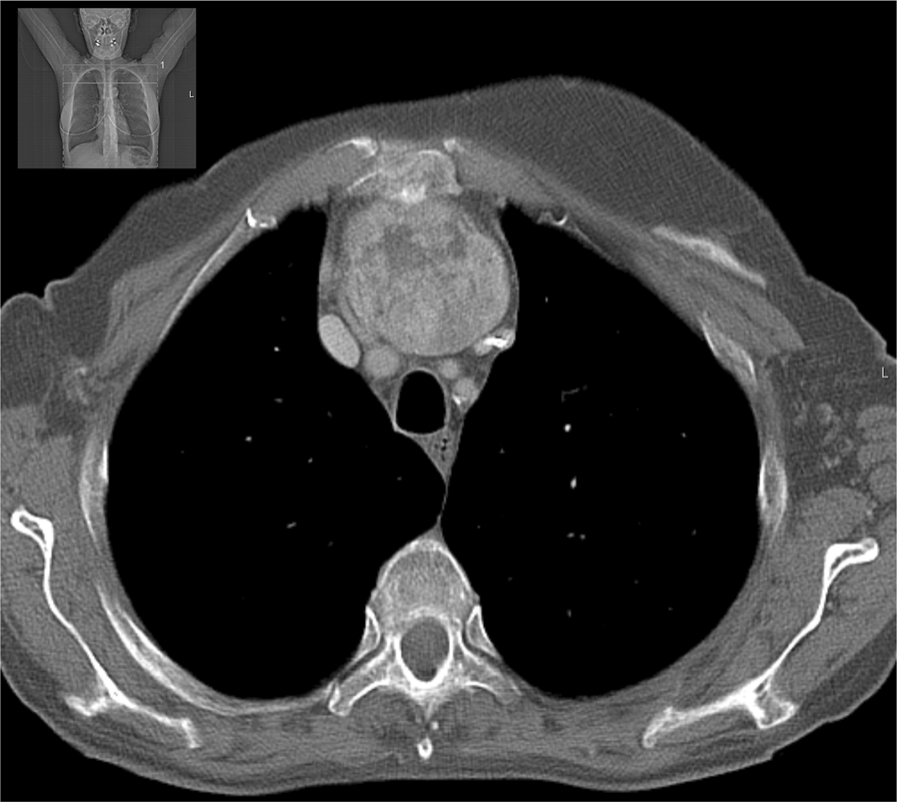
Neck and thorax computed tomography scan showing the heterogeneous 67 × 46 × 52 mm mass in the anterior mediastinum – axial section

**Fig. 3:**
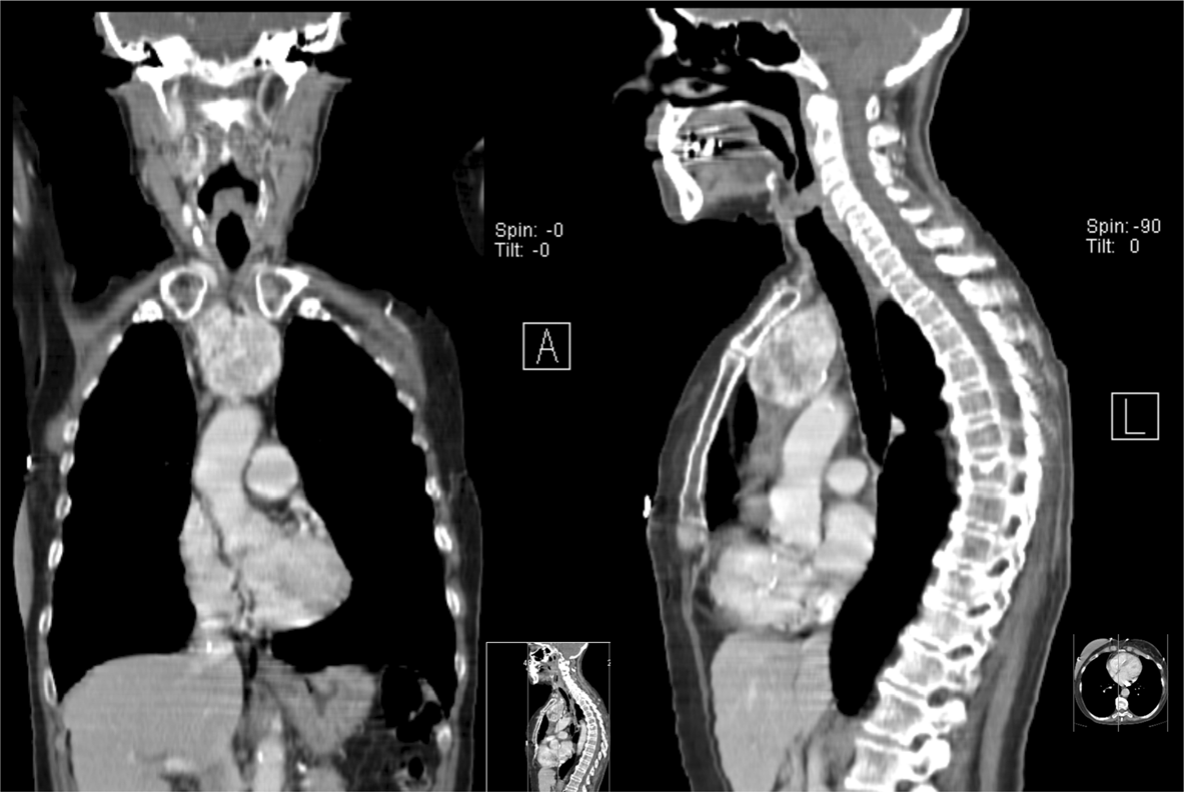
Neck and thorax computed tomography scan – coronal (*left*) and sagittal sections (*right*)

She was admitted to the endocrinology department with a diagnosis of iatrogenic thyrotoxicosis. She was started on bisoprolol 5 mg and levothyroxine was stopped. Her heart rate remained under 100 bpm and her blood pressure was always normal. Her extremities’ rest tremor persisted. She showed no improvement on her free T4 and free T3 levels 10 days after levothyroxine treatment withdrawal (Table 1). Her thyroglobulin levels were increased to 294 mg/mL (0–55). A cervical ultrasound scan revealed no thyroid remnant in the gland bed. Test results for anti-TSH receptor antibodies (TRAb) were positive – 19.7 U/L (<1.8) – and for anti-thyroperoxidase antibodies were negative – 0.6 U/mL (<5.61). Corporal scintigraphy with I-131 with single-photon emission computed tomography (SPECT) revealed an intrathoracic mass (60 × 40 mm) with increased radioiodine uptake and no increased uptake in the thyroid bed (Fig. 1). A computed tomography (CT) scan of her neck and thorax confirmed a heterogeneous mass with 67 × 46 × 52 mm in the anterior mediastinum superiorly (Figs. 2 and 3).

**Table 1:** Evolution of thyroid function

	D1	D10	D13	3 months^a^	8 months^b^
Free T3 (1.71–3.71 pg/mL)	8.46	7.19	9.31	3.02	
Free T4 (0.70–1.48 ng/dL)	3.22	3.19	3.11	1.38	1.42
TSH (0.35–4.94 μU/mL)	0.000			0.002	1.26

*D1* at admission, *D10* 10 days after admission, *D13* 13 days after admission (at discharge), *T3* triiodothyronine, *T4* thyroxine, *TSH* thyroid-stimulating hormone

^a^3 months after discharge and under methimazole 10 mg/day treatment

^b^8 months after discharge and 5 months after surgery and supplemented with levothyroxine 88 μg/day

The final diagnosis was Graves’ disease in mediastinal thyroid tissue. She was discharged with bisoprolol 5 mg/day and methimazole 10 mg/day.

Three months later, after achieving euthyroidism, she underwent surgical resection of the mass through a horizontal cervical incision. The thyroid tissue found in the superior mediastinum had cervical vascularization. There was no complication in the postoperative period. The histology showed hyperfunctional tissue with thyroid follicles of variable shape and form with papillary hyperplasia and some adenomatous areas; no signs of malignancy were found.

Our patient remained asymptomatic and with normal thyroid function under levothyroxine 88 μg/day (1.6 μg/kg) 5 months after surgery.

## Discussion

We present the case of a patient with Graves’s disease in mediastinal thyroid tissue years after total thyroidectomy for a nontoxic multinodular goiter. There are few case reports of Graves’ disease arising from non-normally located thyroid tissue [8–13] and even fewer from thyroid tissue located in the mediastinum [2, 14–18]. In all cases, either Graves’ disease was diagnosed prior to the surgery and then detected in the mediastinum [14, 18]; or the ectopic tissue was first noted during the evaluation of hyperthyroidism [15, 16]. In one case, the ectopic tissue was removed due to compressive symptoms and recurred 9 years later when Graves’ disease developed [17]. To the best of our knowledge, there are no reports of mediastinal thyroid tissue first presenting as hyperthyroidism without previous known history of such condition, neither in truly ectopic tissue nor in a surgical thyroid remnant. The absence of reports of hyperthyroidism in surgical remnants may represent a publication bias: physicians tend to not report eventual surgical failures. Nonetheless, a complete lack of published cases argues for the rarity of this case presentation.

Although the great majority of cases of thyrotoxicosis after total thyroidectomy are caused by excessive levothyroxine therapy, true cases of hyperthyroidism must not be forgotten. Ectopic thyroid tissue or a thyroid remnant may become hyperfunctional, as any thyroid gland. An important clue is the failure of the thyroid hormone levels to normalize after reduction or even withdrawal of levothyroxine as was the case in our patient. Another sign may be an elevated thyroglobulin level. Thyroglobulin is useful to detect residual thyroid tissue after total thyroidectomy and has an important place in the follow-up of patients with differentiated thyroid cancers [19]. Nonetheless, it can be helpful in distinguishing between truly endogenous thyroid production and an exogenous source [1]. Thyroglobulin levels were increased in our patient pointing toward the presence of functional thyroid tissue. Scintigraphy is a very useful method to detect any functional thyroid tissue and its location. In the presented case, whole body scintigraphy revealed a mediastinal mass with increased radioiodine uptake.

The presence of thyroid tissue after total thyroidectomy may correspond to a remnant, ectopic thyroid or metastatic tissue [2]. The presence of a remnant is the most usual cause of residual thyroid tissue [4, 5, 18, 20]. The reported recurrence rate of benign multinodular goiter after total thyroidectomy ranges between as low as 0.33 %, when recurrence is defined by the need of a second surgery [4], to as much as 33 % when based on ultrasonographic examination [5]. A remnant was detected in as many as 93.1 % of patients with differentiated thyroid carcinoma after total thyroidectomy when scintigraphy was performed [20]. A “forgotten goiter” is a portion of the thyroid gland located in the mediastinum missed in the previous total thryroidectomy [2, 18]. This term refers to thyroid tissue that is neither visibly connected to the cervical gland nor truly ectopic tissue. The blood supply of this tissue is usually derived from cervical blood vessels, which makes it an important sign in the differential diagnosis [2, 18]. A remnant of nontoxic multinodular goiter is not clinically relevant most of the time since they are usually small sized [5]. Nevertheless, a remnant may assume clinical significance in the presence of stimulating anti-TSH receptor immunoglobulins and consequent hyperfunction [10].

The presence of ectopic thyroid tissue is rare [6, 21]. In normal embryological development, the thyroid is formed from an endodermal diverticulum in the pharyngeal gut at the 24th day of gestation. It then migrates downward along the midline from the foramen cecum until it reaches its final pretracheal position. Ectopic thyroid tissue results from an abnormal migration along this path [6, 22]. The most common location is the base of the tongue but it may be found as far as the mediastinum or even the abdominal cavity [6]. The mediastinum is a rare location for ectopic thyroid tissue and, when present, it normally coexists with an orthotopic gland and patients are euthyroid [6]. Ectopic thyroid tissue may develop benign or malign pathology usually found in the “normal” thyroid gland [6, 7]. The differentiation between thyroid remnant and ectopic thyroid tissue is not always easy after total thyroidectomy [2].

A thyroid cancer metastasis should always be kept in mind when thyroid tissue is identified in a non-normal location [6].

In the particular case of our patient, it is possible that the non-normally located thyroid tissue may have gone unnoticed while still functioning normally. The diagnosis of hyperfunction associated with the development of Graves’ disease was further delayed because our patient was on levothyroxine supplementation. Malignant cells were not found on histological examination, excluding the hypothesis of neoplasia. Whether the mediastinal thyroid tissue found in our patient corresponds to truly ectopic tissue with no attachment to the orthotopic thyroid gland, or represents thyroid tissue not removed in the previous surgery that increased in size under stimulation of the anti-TSH receptors antibodies with the onset of the autoimmune disease remains unknown, but the presence of cervical vascularization argues in favor of the latter [6]. The decision between surgical or radioiodine treatment was made in accordance with our patient’s personal preferences.

## Conclusions

Although thyrotoxicosis after total thyroidectomy is mostly due to excessive supplementation, physicians should keep in mind the possibility of true hyperthyroidism. The presence of thyroid tissue after total thyroidectomy in our patient may correspond to a remnant or, less likely, ectopic thyroid tissue that became hyperfunctional in the presence of TRAbs.

## Consent

Written informed consent was obtained from the patient for publication of this case report and any accompanying images. A copy of the written consent is available for review by the Editor-in-Chief of this journal.

## Abbreviations

CT: computed tomography
SPECT: single-photon emission computed tomography
T3: triiodothyronine
T4: thyroxine
TRAb: anti-TSH receptor antibody
TSH: thyroid-stimulating hormone
